# Novel and recurrent *FBN1* mutations causing Marfan syndrome in two Chinese families

**DOI:** 10.3389/fmed.2022.1086844

**Published:** 2022-12-13

**Authors:** Dandan Li, Jun Qiao, Dandan Huang, Ruru Guo, Jian Ji, Wei Liu

**Affiliations:** ^1^Department of Ophthalmology, Tianjin TEDA Hospital, Tianjin, China; ^2^Department of Ophthalmology, Lanzhou Huaxia Eye Hospital, Lanzhou, Gansu, China; ^3^Department of Ophthalmology, Taihe Hospital, Hubei University of Medicine, Shiyan, Hubei, China; ^4^Tianjin Key Laboratory of Retinal Functions and Diseases, Tianjin Branch of National Clinical Research Center for Ocular Disease, Eye Institute and School of Optometry, Tianjin Medical University Eye Hospital, Tianjin, China

**Keywords:** Marfan syndrome, *FBN1*, bioinformatics analysis, mutation, phenotype

## Abstract

**Background:**

To explore the genetic defects of two families with autosomal dominant Marfan syndrome (MFS).

**Methods:**

Two families with MFS were enrolled in this study. The detailed ocular presentations of the patients were recorded. Whole exome sequencing was performed to explore the pathogenic variants and Sanger sequencing was performed to confirm the gene mutations. Segregation analysis among the family members was made and bioinformatics analysis was performed to predict the functional impact of the mutations.

**Results:**

The main ocular presentations of the probands were increased axial length and ectopia lentis. Using whole exome sequencing and Sanger sequencing, a novel heterozygous missense mutation (c.5060G > C, p.Cys1687Ser) and a recurrent missense mutation (c.2168A > T, p.Asp723Val) were identified within *FBN1*, which were co-segregated with the MFS phenotype in the families. Evolutionary conservation analysis showed that codons 723 and 1,687 were highly conserved among several species. Functional impact predictions made using several online programs suggested that the mutations were pathogenic.

**Conclusion:**

We identified a novel and a recurrent missense mutation in *FBN1* in two Chinese families with MFS using whole exome sequencing, and our bioinformatics analysis indicated that the mutations were disease-causing. Our results expand the mutation spectrum of *FBN1* and could help us better understand the genetic defects of the patients with MFS.

## 1 Introduction

Marfan syndrome (MFS) is a rare disorder that affects the connective tissues of the body. It mainly involves the ocular, cardiovascular, and musculoskeletal systems, and has diverse clinical presentations. The incidence of MFS is about 2–3 per 10,000 individuals (1). MFS is autosomal dominant inherited, and *FBN1* is implicated in most MFS cases. *FBN1*, located on 15q15-q21.1, comprises 65 exons and encodes the 2,871-amino acid fibrillin-1 protein (2), which is widely expressed in the aorta, tendons, periosteum of the bones, and ciliary zonules of the eye (3). Although the risk of developing MFS is higher in patients with a family history, the condition does not discriminate between different genders and ethnicities (1, 2). In a cohort of 131 Chinese patients with MFS, mutations in *FBN1* were detected in 82 patients (4), suggesting that *FNB1* mutations are the predominant cause of MFS. Although several mutations in *FBN1* have been reported to be responsible for MFS, the exact phenotype-genotype correlation remains unclear (3, 4).

In this study, we investigated two Chinese families with MFS. We identified a novel missense mutation (c.5060G > C, p.Cys1687Ser) and a recurrent missense mutation (c.2168A > T, p.Asp723Val) in *FBN1* using whole exome sequencing and Sanger sequencing.

## 2 Materials and methods

### 2.1 Patients

This study was approved by the ethics committee of Tianjin Medical University Eye Hospital (2021-KY03) and followed the tenets of the Declaration of Helsinki. Two Chinese families were enrolled, and written informed consent was obtained from the participants. A peripheral venous blood sample was collected from each enrolled family member for further analysis.

### 2.2 Whole exome sequencing and bioinformatics analysis

The whole exome sequencing and data analysis procedures were previously reported (5–7). Briefly, we first extracted genomic DNA from the blood samples according to the standard procedure of the manufacturer (MagPure Buffy Coat DNA Midi KF Kit, Magen, China), and the qualified genomic DNA was then sequenced with PE100 + 100 on MGISEQ-2000. We applied the BGI MGIEasy V4 chip, which contains exons of all human genes and their adjacent ± 20 bp introns, to capture the targeted sequences. Bioinformatics processing and data analysis were then performed to explore the potential variants after we received the primary sequencing data. Several databases, including the 1,000 Genomes Project, HapMap, NCBI dbSNP, and a database of 200 normal Chinese adults, were used to filter and estimate all the SNVs and indels. Finally, Sanger sequencing was used to validate all mutations and potential pathogenic variants. The Human Gene Mutation Database (HGMD)^1^ was introduced to screen previously reported mutations. To rule out the possibility of a polymorphism, the mutations were also blasted in the 1,000 Genomes Project,^2^ ExAC,^3^ HapMap,^4^ ESP6500,^5^ NCBI dbSNP,^6^ GnomAD,^7^ and a database of 200 normal Chinese adults.

### 2.3 Functional impact prediction

Calibrated predictions of the possible functional impacts of the *FBN1* mutations were made using several online programs, including VARSOME,^8^ PolyPhen2,^9^ PROVEAN,^10^ and MutationTaster,^11^ etc. Based on the standards proposed by the American College of Medical Genetics and Genomics (ACMG) (8), the variants were also defined as “likely benign or benign,” “of uncertain significance,” or “likely pathogenic or pathogenic.”

### 2.4 Evolutionary conservation analysis

Evolutionary conservation analyses among several species were performed using Clustal Omega.^12^

### 2.5 Protein secondary structure analysis

Secondary structure analyses of the wild-type and the mutant proteins were performed using Network Protein Sequence Analysis.^13^

### 2.6 Protein structural effect evaluation

Protein structure homology modeling and protein structural effect evaluation were performed using HOPE^14^ (9). Firstly, UniProt ID of P35555 was input to obtain the amino acid sequence of *FBN1*. The residues and mutations were then selected to finalize the analysis.

## 3 Results

### 3.1 Clinical evaluation

**FIGURE 1:**
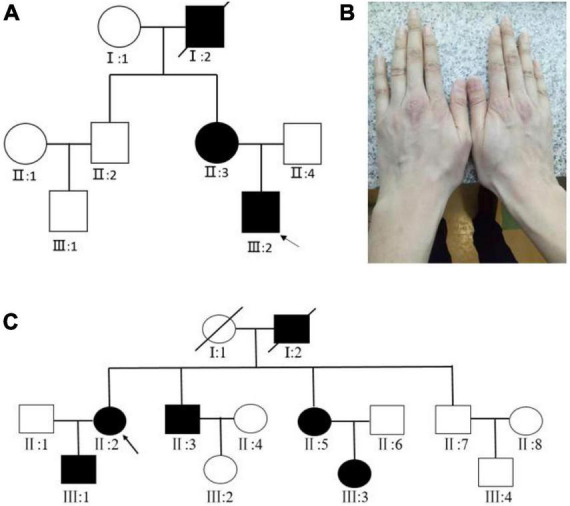
**(A)** Pedigree map of Family 1. The arrow indicates the proband. Squares and circles symbolize males and females, respectively. Black and white denote affected and unaffected individuals, respectively. **(B)** The long fingers of the proband of Family 1. **(C)** Pedigree map of Family 2.

For Family 1, two patients (II:3, III:2) and one normal individual (II:4) were enrolled in our study (Figure 1A). The proband (III:2) was 30 years old and had been diagnosed with MFS in his childhood. On presentation, the proband was tall and had long fingers (Figure 1B). The vision was 20/200 in both eyes. In the right eye and left eye, the axial length was 30.61 and 34.01 mm, the steep K was 40.26 D and 40.74 D, the flat K was 39.06 D and 38.75 D, and the intraocular pressure was 17.8 and 15.7 mmHg, respectively. The medical records of the proband were reviewed. The patient accepted bilateral clear lens extraction because of bilateral nasal superiorly dislocated lens when he was 14 years old. Three months after the surgery, retinal detachment occurred in the right eye, and he underwent vitrectomy and silicone oil tamponade. The oil in the right eye was removed 6 months later. His mother and grandfather were diagnosed with MFS at early ages. His mother (II:3) was 53 years old and underwent clear lens extraction because of ectopia lentis when she was young. His mother’s vision was 20/200 and 20/300, her intraocular pressure was 20.7 and 19.9 mmHg, and her axial length was 28.45 and 27.87 mm in the right eye and left eye, respectively. There was no consanguineous marriage in this family.

For Family 2, four affected (III:1, II:2, II:3, III:5) and three unaffected individuals (III:4, II:1, II:7) were enrolled in our study (Figure 1C). The proband (II:2) was 50 years old, and she was diagnosed with MFS when she was six. The patient had accepted bilateral clear lens extraction for bilateral temporally dislocated lens 10 years ago. On presentation, her visual acuity was finger-counting and 20/200, her best-corrected visual acuity was 20/200 and 20/50, her axial length was 27.15 and 28.10 mm, and her intraocular pressure was 18.9 and 19.1 mmHg for the right eye and left eye, respectively. Her son (III:1) was 25 years old and was diagnosed with MFS when he was five. He also underwent bilateral clear lens extraction for ectopia lentis several years ago. His visual acuity was 20/100 and 20/80, his axial length was 25.23 and 26.37 mm, and his intraocular pressure was 15.1 and 13.5 mmHg for the right eye and left eye, respectively. The other two enrolled patients in this family (II:3, II:5) reported that they both had high myopia and had accepted surgery for ectopia lentis, but unfortunately, the detailed clinical data were not available.

### 3.2 Mutation identification in *FBN1*

**FIGURE 2:**
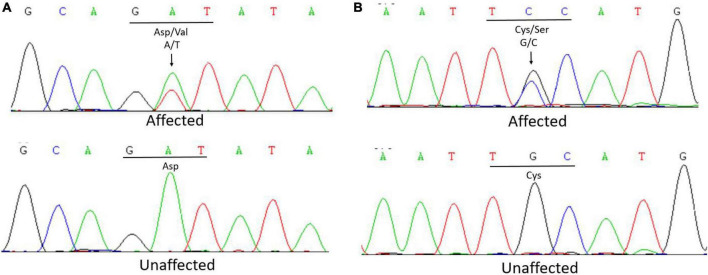
**(A)** Sanger sequencing of *FBN1* detected a c.2168A > T transversion in affected patients that caused the replacement of a wild-type aspartic acid with valine at codon 723 in Family 1. **(B)** Sanger sequencing of *FBN1* detected a c.5060G > C transversion in affected patients that caused the replacement of a wild-type cysteine with serine at codon 1,687 in Family 2.

Whole exome sequencing of the proband of family 1 (III:2) revealed a heterozygous transversion in exon 19 (c.2168A > T) of *FBN1*, which has been reported previously (10). The mutation changes wild-type aspartic acid to valine at codon 723 (p.Asp723Val; Figure 2A). The mutation was found in all affected individuals (II:3, II:2) but was not detected in the unaffected individual (II:4) in this family using further Sanger sequencing.

In Family 2, the whole exome sequencing of the proband revealed a novel heterozygous transversion in exon 41 (c.5060G > C) of *FBN1*. This mutation changes a wild-type cysteine to serine at codon 1,687 (p.Cys1687Ser; Figure 2B). Using Sanger sequencing, the mutation was found in all affected individuals (II:3, II:5, III:1) and was not detected in the unaffected individuals (II:1, II:7, III:4) in this family, indicating that the mutation was co-segregated with the phenotype. The mutation was not found in the 1,000 Genomes Project, ExAC, GnomAD-EAS_exome_ALL, HapMap, ESP6500, NCBI dbSNP, GnomAD_exome_ALL, or the database of 200 normal Chinese adults.

No other rare variants classified as pathogenic or likely pathogenic were identified in the other genes (*FBN2*, *TGFBR1*, *TGFBR2*, *LTBP-1*, *LTBP-2*, *LTBP-3*, *SKI*, etc.) related to MFS in either family.

### 3.3 Functional impact prediction

Most of the online bioinformatics programs produced a result of “Pathogenic” (Table 1). According to ACMG guidelines and standards, the c.2168A > T, p.Asp723Val mutation was defined as “Pathogenic” (PM5 + PP3 + PM1 + PM2 + PP5) and the c.5060G > C, p.Cys1687Ser mutation as “Likely pathogenic” (PP3 + PM5 + PM1 + PM2).

**TABLE 1:** Calibrated prediction of the possible functional impact of the *FBN1* mutations by online programs.

Program	c.2168A > T, p.Asp723Val	c.5060G > C, p.Cys1687Ser
BayesDel addAF	Pathogenic (0.567)	Pathogenic (0.5789)
BayesDel noAF	Pathogenic (0.5767)	Pathogenic (0.5937)
MetaLR	Pathogenic (0.9807)	Pathogenic (0.9897)
MetaRNN	Pathogenic (0.992)	Pathogenic (0.9925)
MetaSVM	Pathogenic (1.0588)	Pathogenic (1.0013)
REVEL	Pathogenic (0.985)	Pathogenic (0.957)
BLOSUM	Uncertain (8)	Uncertain (3)
DANN	Uncertain (0.9914)	Uncertain (0.9941)
EIGEN	Pathogenic (0.9374)	Pathogenic (1.0099)
EIGEN PC	Pathogenic (0.8718)	Pathogenic (0.9319)
FATHMM	Pathogenic (5.39)	Pathogenic (5.89)
FATHMM-MKL	Pathogenic (0.987)	Pathogenic (0.9898)
FATHMM-XF	Pathogenic (0.9378)	Pathogenic (0.9751)
LIST-S2	Uncertain (0.9571)	Uncertain (0.9211)
LRT	Pathogenic (0)	Pathogenic (0)
M-CAP	Pathogenic (0.8507)	Pathogenic (0.9575)
MutationTaster	Uncertain (1)	Uncertain (1)
MutPred	Pathogenic (0.942)	Pathogenic (0.94)
MVP	Pathogenic (0.9951)	Pathogenic (0.9708)
Polyphen2	Pathogenic (0.999)	Pathogenic (0.987)
PrimateAI	Uncertain (0.7547)	Pathogenic (0.8538)
PROVEAN	Pathogenic (7.857)	Pathogenic (9.012)
SIFT	Pathogenic (0)	Pathogenic (0)
SIFT4G	Pathogenic (–0.001)	Pathogenic (0)

### 3.4 Evolutionary conservation analysis

**FIGURE 3:**
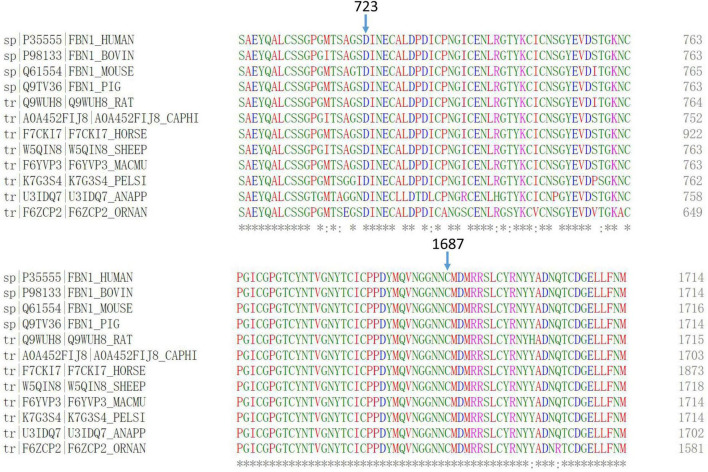
Multiple-sequence alignments of *FBN1* showed that codons 723 and 1,687 were highly conserved among several species.

Evolutionary conservation analysis revealed that codons 723 and 1,687 were located within a highly conserved region among several species (Figure 3).

### 3.5 Protein secondary structure analysis

**FIGURE 4:**
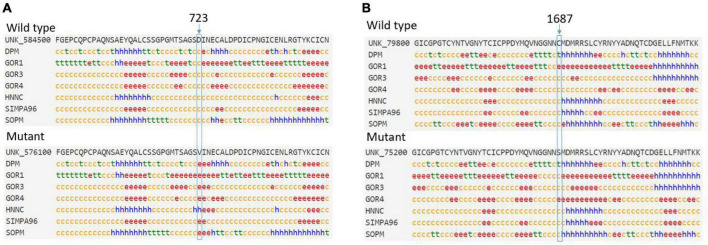
**(A)** The predicted secondary structure indicated that an original random coil in the wild-type *FBN1* was replaced by an extended strand or alpha helix in the p.Asp723Val mutant FBN1. **(B)** The p.Cys1687Ser mutant did not cause much change in the predicted secondary structure. c, random coil; e, extended strand; h, alpha helix; t, beta turn.

The predicted secondary structure indicated that an original, flexible, random coil in the wild-type *FBN1* was replaced by a more stable extended strand or alpha helix in the p.Asp723Val mutant *FBN1* (Figure 4A). However, the mutant p.Cys1687Ser did not cause much change to the predicted secondary structure (Figure 4B).

### 3.6 Protein structural effect evaluation

**FIGURE 5:**
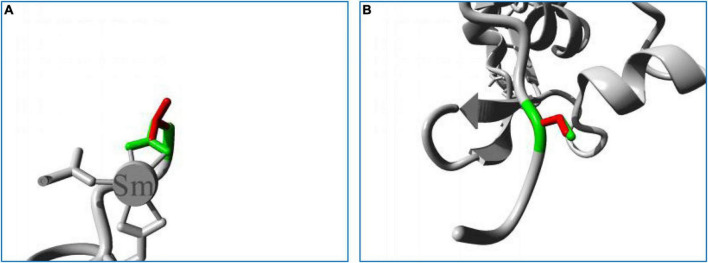
Close-up image of the superimposed structures of wild-type and mutant residues. **(A)** Asp723Val. **(B)** Cys1687Ser. The protein core is shown in gray, while the amino acid side chains of the wild-type (green) and the mutant (red) residues are represented as sticks.

In structure homology modeling, the p.Asp723Val mutation changed the side chain of the protein (Figure 5A). The Asp723 residue is involved in a metal-ion (Sm) contact (Figure 5A), and the differences in both size and charge caused by mutation can make the metal-ion interactions of Asp723 less stable. The hydrophobic difference can further interfere with the formation of hydrogen bonds, and the mutation might also disturb the function of the protein by interfering with the normal interaction of Asp723 with other molecules.

The p.Cys1687Ser mutation also changed the side chain of the protein (Figure 5B). The replacement of Cys with Ser can sever the bonds formed by cysteines, impairing the stability of the protein, and impede the normal hydrophobic interactions of Cys with other molecules on the surface of the protein. The Cys1687 residue is located within an Egf-like calcium-binding domain, and the p.Cys1687Ser mutation can disturb this domain and affect its function by introducing an amino acid with different properties.

Together, all these observations indicate that the mutations found in this study were pathogenic and disease-causing.

## 4 Discussion

In this study, we evaluated two Chinese families with MFS, and the main ocular presentations were increased axial length and ectopia lentis. We also identified a novel missense mutation (c.5060G > C, p.Cys1687Ser) and a recurrent missense mutation (c.2168A > T, p.Asp723Val) in *FBN1* using whole exome sequencing and Sanger sequencing, and our bioinformatics analysis indicated that both the mutations were disease-causing.

Marfan syndrome is a rare disease with manifestations mainly involving the ocular, musculoskeletal, and cardiovascular systems. Cardiovascular manifestations represent the major morbidity and mortality factors, with aortic root dissection being the main cause of death in MFS patients. Other cardiovascular manifestations include left ventricular dilation, mitral valve prolapse, and pulmonary artery enlargement (11–14). The main musculoskeletal presentation of MFS is overgrowth of the long bones (15), which leads to altered ratios among the body’s segments (overgrowth of arms and legs), anterior chest deformity (overgrowth of the ribs), and arachnodactyly (overgrowth of the fingers) (1). The typical ocular features of MFS are ectopia lentis and myopia, consistent with the results of our study. Ectopia lentis has been shown to occur in 45–87% of patients with MFS (16–18) and is a major criterion for diagnosis (19). The lens dislocation is resulted from the insufficiency of the ciliary zonules. Lens dislocation in MFS can occur in any direction, but typically superiorly (3), as demonstrated in our study. Myopia is another common ocular presentation in patients with MFS, which often begins at an early age and has a rapid progression. Other manifestations of the ocular system include an elongated globe, an abnormally flat cornea, hypoplasia of the ciliary muscle and iris, amaurosis, an increased risk of early cataract and glaucoma, and a predisposition for retinal detachment (19–21). In patients who have not developed typical cardiovascular symptoms, ocular symptoms may comprise the initial presentations of MFS, necessitating a more comprehensive diagnostic workup.

Up to now, about 3,000 different *FBN1* mutations for MFS have been included in the UMD-FBN1 database.^15^ Although so many *FBN1* mutations have been reported to be responsible for MFS, the exact phenotype-genotype correlation in MFS is still unclear because of the inter- and intra-family clinical variability. However, a few phenotype-genotype relationships have been firmly established so far, such as mutations in exons 24–32 of *FBN1* being associated with neonatal MFS and a severer phenotype (22–24), while mutations in exons 43–65 have been associated with a substantial increase in cardiovascular manifestations (25–27). Meanwhile, patients with haploinsufficiency mutations tended to have more severe cardiovascular involvements than patients with dominant negative mutations (28). For ocular manifestations, in a large cohort including 1,013 probands with pathogenic *FBN1* mutations, missense mutations producing or substituting cysteines were found to be related with more frequent ectopia lentis when compared with other missense mutations (22). Mutations introducing premature termination codons were connected to severer skin and skeletal presentations but less common ectopia lentis and retinal detachment (3, 22, 23, 29). This is in accordance with the results of our study; lens dislocation was the major ocular manifestation of the proband with the p.Cys1687Ser mutation in Family 2. Two other missense mutations in codon 1,687 have been reported previously: p.Cys1687Arg (30) and p.Cys1687Phe (31). The patient who carried the p.Cys1687Arg mutation was diagnosed with incomplete MFS, but the detailed clinical data were not available (30). The patient with the p.Cys1687Phe mutation was a 3-year-old Caucasian boy whose ocular presentation was mainly ectopia lentis (31). However, although the mutation identified in Family 1 (p.Asp723Val) was located in exon 19 and was not a cysteine mutation, the patient presented with bilateral ectopia lentis, myopia, and retinal detachment, indicating the highly heterogeneous nature of MFS. The p.Asp723Val mutation was first reported in a German 8-year-old girl whose ocular involvement also included ectopia lentis and myopia (10). All of these suggest that the phenotype-genotype correlations of MFS are not yet clear and need further study to confirm.

Although our bioinformatics analysis indicated that the mutations detected in our study were disease-causing, the exact molecular pathogenesis of MFS remains unknown (1, 2). A traditional dominant-negative mechanism has been proposed to be implicated in the pathogenesis of MFS. In a dominant-negative model, Eldadah et al. illustrated that an experimentally introduced mutant *FBN1* allele in the presence of two endogenous wild-type alleles is sufficient to reproduce the MFS cellular phenotype by reducing fibrillin-1 accumulation in the extracellular matrix and disrupting normal microfibrillar assembly (32). On the other hand, in a transgenic mice model, Judge et al. demonstrated that selected mutations (e.g., C1039G) caused a disorganized microfibril architecture and that the introduction of exogenous wild-type fibrillin-1 can rescue phenotypes associated with the C1039G mutation (33), indicating that wild-type fibrillin-1 haploinsufficiency, rather than mutant proteins production, might be the predominant determinant of failed microfibrillar assembly.

## 5 Conclusion

In this study, we evaluated two Chinese families with MFS whose main ocular presentations were increased axial length and ectopia lentis. Using whole exome sequencing and Sanger sequencing, we identified a novel missense mutation (c.5060G > C, p.Cys1687Ser) and a recurrent missense mutation (c.2168A > T, p.Asp723Val) in *FBN1*, and our bioinformatics analysis indicated that both the mutations were disease-causing. Our results expanded the mutation spectrum of *FBN1* and could help broaden the phenotype-genotype relationships of MFS. However, the exact disease-causing mechanism of the mutations needs further functional experiments to confirm.

## Data availability statement

The genetic sequencing data in this manuscript were not deposited to a public repository to protect patient privacy/confidentiality. The datasets used and/or analyzed during the current study are available from the corresponding author on reasonable request.

## Ethics statement

The studies involving human participants were reviewed and approved by the Ethics Committee of Tianjin Medical University Eye Hospital. The patients/participants provided their written informed consent to participate in this study. Written informed consent was obtained from the individual(s) for the publication of any potentially identifiable images or data included in this article.

## Author contributions

WL and JJ designed and supervised the study. JQ collected the pedigree. DL and RG drafted the manuscript. WL, RG, DH, and JJ analyzed the data. All authors read and approved the final manuscript.

## Footnotes

1
http://www.hgmd.cf.ac.uk/ac/index.php

2
https://www.internationalgenome.org/data

3
https://gnomad.broadinstitute.org/

4
http://hapmap.ncbi.nlm.nih.gov

5
http://evs.gs.washington.edu/EVS/

6
https://www.ncbi.nlm.nih.gov/snp/

7
http://www.gnomad-sg.org/

8
http://varsome.com

9
http://genetics.bwh.harvard.edu/pph2/

10
http://provean.jcvi.org/index.php

11
http://mutationtaster.org/

12
https://www.ebi.ac.uk/Tools/msa/clustalo/

13
https://npsa-prabi.ibcp.fr/cgi-bin/npsa_automat.pl?page=/NPSA/npsa_seccons.html

14
https://www3.cmbi.umcn.nl/hope/

15
http://www.umd.be/
